# Peripheral serotonin-mediated system suppresses bone development and regeneration via serotonin 6 G-protein-coupled receptor

**DOI:** 10.1038/srep30985

**Published:** 2016-09-01

**Authors:** Hyung-Mun Yun, Kyung-Ran Park, Jin Tae Hong, Eun-Cheol Kim

**Affiliations:** 1 Department of Oral and Maxillofacial Pathology, School of Dentistry, Kyung Hee University, Seoul 130-701, Republic of Korea; 2 Department of Oral & Maxillofacial Regeneration, Graduate School, Kyung Hee University, Seoul 130-701, Republic of Korea; 3 College of Pharmacy and MRC, Chungbuk National University, 12 Gaesin-dong, Heungduk-gu, Cheongju, Chungbuk 361-763, Korea; 4 Department of Oral and Maxillofacial Pathology, School of Dentistry and Research Center for Tooth & Periodontal Regeneration (MRC), Kyung Hee University, Seoul 130-701, Republic of Korea.

## Abstract

Serotonin is important in brain functions and involved in neurological diseases. It is also drawn considerable attention in bone disease since it mainly produced by the gut. Serotonin 6 G-protein-coupled receptor (5-HT_6_R) is clinical targets for the treatment of neurological diseases. However, 5-HT_6_R as a therapeutic target in bone has not been reported. Herein, we found that 5-HT_6_R showed higher expression in bone, and its expression was increased during bone remodeling and osteoblast differentiation. The activation of 5-HT_6_R by ST1936 caused the inhibition of ALP activity and mineralization in primary osteoblast cultures, which was antagonized by SB258585, an antagonist and by the knockdown of 5-HT_6_R. Further investigation indicated that 5-HT_6_R inhibited osteoblast differentiation via Jab1 in BMP2 signaling but not PKA and ERK1/2. *In vivo* studies showed that the activation of 5-HT_6_R inhibited bone regeneration in the calvarial defect mice and also delayed bone development in newborn mice; this response was antagonized by SB258585. Therefore, our findings indicate a key role of 5-HT_6_R in bone formation through serotonin originating in the peripheral system, and suggest that it is a novel therapeutic target for drug development in the bone repair and bone diseases.

Serotonin is important in the regulation of virtually all brain functions, and strongly implicated in psychiatric and neurological diseases such as Alzheimer’s disease and depression^1^. However, most serotonin is found outside of the central nervous system and is mainly synthesized in the gut by tryptophan hydroxylase-1 (Tph-1)^2^. 95% of total body serotonin is released into the gut by enterochromaffin cells and enter into blood circulation, indicating that the serotonin system has various functions beyond actions of serotonin as a well-known neurotransmitter^3^,^4^.

The recent research was reported that a serotonin-mediated system originating in the gut can regulate bone mass^5^,^6^. Selective serotonin reuptake inhibitors (SSRIs) reduced bone mass in mice^7^ and also clinical data showed a significant increase for fractures in patients treated with SSRIs^8^. Yadav *et al*. found that serotonin production in the gut can enter into blood circulation and reduce bone formation^5^. Reporting in *Nature medicine*, Yadav *et al*. also reported that pharmacological inhibition of gut-derived serotonin synthesis by LP533401, a small molecule inhibitor of Tph-1 is a potential therapeutic treatment for low–bone-mass diseases^9^.

The physiological diversity of serotonin system is mediated by multiple serotonin receptors (5-HTRs), which have been divided into seven classes (5-HT_1_R through 5-HT_7_R) on the basis of their signaling pathway^10^. Among them, 5-HT_6_R, a G-protein-coupled receptor (GPCR) is one of the latest cloned receptor and positively coupled to adenylyl cyclase via stimulatory G (Gs) proteins and increases intracellular cyclic AMP (cAMP) production by the activation of adenylyl cyclase^11^. It was previously demonstrated that 5-HT_6_R physically and functionally interacts with various proteins including Fyn tyrosine kinase and Jun activation domain-binding protein 1 (Jab1)^12^,^13^. Over the past decades, the 5-HT_6_R has gained increasing attention in the brain and has become a clinical target for treating neurological diseases such as Alzheimer’s diseases, depression, and schizophrenia^14^,^15^,^16^. However, there is no study for 5-HT_6_R in the peripheral organs.

In the present study, we investigated the relative expression of 5-HT_6_R in bone compared to brain, and examined its role in *in vitro* osteoblast differentiation and *in vivo* bone regeneration and bone development via peripheral serotonin system. Our findings indicate that 5-HT_6_R has a crucial role in bone metabolism and will shed light on how peripheral serotonin system regulates bone metabolism.

## Results and Discussion

**Figure 1:**
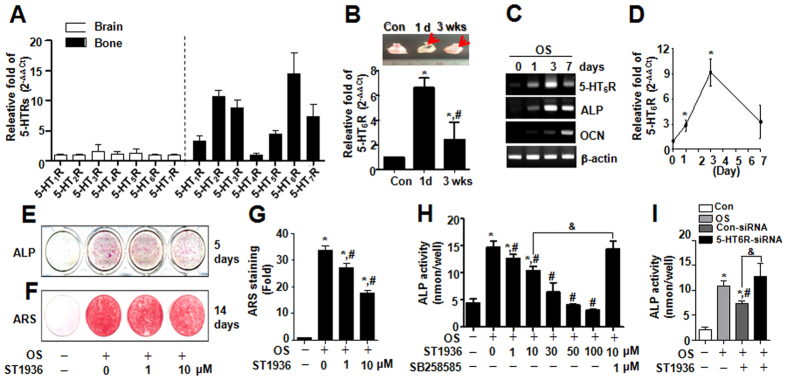
5-HT_6_R is highly expressed in bone and its activation attenuated *in vitro* osteoblast differentiation in primary calvarial osteoblasts. (**A**) After total RNA was isolated from brain and bone in female 10-week-old ICR mice, the expression levels of serotonin receptors were analyzed by real-time PCR and normalized to that of β-actin. (**B**) Calvarial defects were created in female 10-week-old ICR mice. Arrowhead indicates the defect region (*upper*). The mRNA level of 5-HT_6_R at 1 day and 3 weeks was analyzed by real-time PCR and normalized to that of β-actin (*bottom*). (**C,D**) After primary calvarial osteoblasts from 1-day-old ICR mice were cultured with osteogenic supplement medium (OS) for 0, 1, 3, and 7 days, the mRNA level analyzed by RT-PCR (**C**) or real-time PCR (**D**) and was normalized to that of β-actin. (**E**–**G**) Cells were treated with OS in the presence of 1 and 10 μM ST1936 for 5 or 14 days. Differentiation was assessed by ALP staining (**E**), and Alizarin Red staining (**F**). The intensity of Alizarin Red staining was determined by optical density (**G**). (**H,I**) ALP activity was analyzed in the presence of the indicated concentrations of ST1936 and 1 μM SB258585 for 5 days (**H**) or analyzed in the presence of 10 μM ST1936 for 5 days, 24 h after transfection with 5-HT_6_R-siRNA (**I**). Data shown represent the means ± SEM of three independent experiments. *p < 0.05, vs. control. ^#^*p* < 0.05, vs. OS. &, *p* < 0.05, vs. 10 μM SB258585 or vs. 5-HT_6_R siRNA with 10 μM ST1936.

In an effort to elucidate how peripheral serotonin system regulates bone formation, we first examined the relative expression of repective 5-HTRs between the bone and brain. Our results showed that 5-HT_6_R showed relatively higher expression in bone compared to brain (Fig. 1A). Given that 5-HT_6_R has been gaining great attention for treating neurological diseases in brain^14^,^17^,^18^, it is very important to elucidate the fundamental mechanisms responsible for peripheral serotonin-mediated bone formation via 5-HT_6_R.

Following critical-sized calvarial bone defects, the expression level of 5-HT_6_R increased in the early stage of differentiation or remodeling (Fig. 1B) and also the 5-HT_6_R mRNA level was upregulated in a time-dependent manner and followed by a decrease (Fig. 1C,D), suggesting that 5-HT_6_R might have regulatory or tuning roles to prevent abnormal osteoblast differentiation.

Primary osteoblast cultures from newborn mouse calvaria are capable of differentiating *in vitro* into mature osteoblasts that is characterized by increased expression of the early marker alkaline phosphatase (ALP), later marker osteocalcin (OCN), and mineralization^19^. To examine the role of 5-HT_6_R in osteoblast differentiation and function *in vitro*, primary osteoblasts were cultured and treated with a recently developed selective 5-HT_6_R agonist, ST1936^20^. The activation effects of 5-HT_6_R were determined by ALP activity and the formation of mineralized bone nodules. ALP staining (Fig. 1E) and mineralization (Fig. 1F,G) were decreased with ST1936 treatment in a concentration-dependent manner. The effects of 5-HT_6_R was antagonized by SB258585, a specific antagonist (Fig. 1H) and attenuated by the knockdown of endogenous 5-HT_6_R (Fig. 1I), indicating that 5-HT_6_R is involved in osteoblast differentiation for serotonin-mediated bone formation.

**Figure 2:**
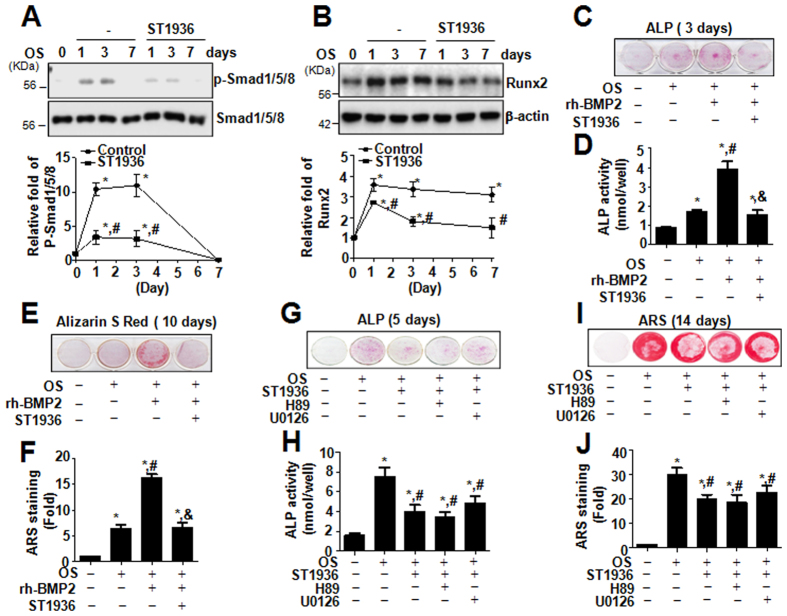
5-HT_6_R inhibits osteoblast differentiation via the BMP-2 signaling pathway but not Gs-protein signaling. (**A,B**) Primary calvarial osteoblasts from 1-day-old ICR mice were cultured with OS in the presence of 10 μM ST1936 for 0, 1, 3, and 7 days. Phospho-Smad1/5/8 and Smad1/5/8 (**A**), and Runx2 (**B**) were assessed by Western blot analysis. **(C**–**F)** Cells were cultured in OS with rh-BMP2 in the presence of ST1936 for 3 (**C,D**) and 10 days (**E,F**). ALP activity was measured via ALP staining (**C**) and ALP activity (**D**). Mineralized nodule formation was assessed by Alizarin Red staining (**E**), and then optical density was measured at 590 nm. (**F**). **(G–J**) Cells were pretreated with H89 (1 μM) or U0126 (1 μM) for 1 h, and then cultured in OS with ST1936 for 5 days (**G**,**H**) and 14 days (**I,J**). ALP activity was measured via ALP staining (**G**) and ALP activity (**H**). Mineralized nodule formation was assessed by Alizarin red staining (**I**) and the stains were eluted and measured at 590 nm. (**J**). Data shown represent the means ± SEM. *p < 0.05, vs. control. ^#^*p* < 0.05, vs. OS. &, *p* < 0.05, vs. ST1936.

The bone morphogenetic protein-2 (BMP-2) signaling pathway plays an important role in osteoblast differentiation and subsequent bone formation or remodeling^21^. Canonical BMP-2 signaling occurs through the phosphorylation of Smad1/5/8 and induces subsequent activation of the transcription of bone-specific genes, leading to bone formation^22^. To determine the mechanism underlying the effect of 5-HT_6_R in osteoblast differentiation, the effect of 5-HT_6_R on BMP-2 signaling was examined in primary calvarial osteoblasts. Our data demonstrated that the treatment with ST1936 attenuated the phosphorylation of Smad1/5/8, the central molecules in BMP-2 signaling (Fig. 2A), and decreased the expression of Runx2, the downstream target gene of BMP-2 signaling (Fig. 2B). To further understand the role of 5-HT_6_R in osteogenic differentiation, rh-BMP-2 was treated with ST1936 in primary calvarial osteoblasts. Rh-BMP-2-induced ALP activity and mineralized nodule formation in primary osteoblasts were suppressed dramatically by ST1936 (Fig. 2C–F). Thus, these results suggested that the BMP-2 signaling pathway has an important role in 5-HT_6_R-mediated differentiation and function of osteoblasts.

5-HT_6_R is a GPCR that increases cAMP by stimulating adenylyl cyclase, leading to the activation of PKA^18^. These GPCRs typically act through Gs proteins in response to extracellular stimuli including growth factors and neurotransmitters^23^. We firstly reported the cellular mechanisms of 5-HT_6_R-mediated Gs protein signaling via Ras-Raf-MEK-ERK1/2 pathway, providing new insights into the physiological roles of 5-HT_6_R in brain^12^,^13^,^24^. However, we, herein, observed that the differentiation of primary osteoblasts is not related to the Gs protein signaling of 5-HT_6_R (the PKA and ERK1/2 pathway) (Fig. 2G–J). Similar to our observation, reporting in *Nature Chem Biol*, Seo *et al*.^23^, and Duhr *et al*.^25^, also described that 5-HT_6_R controls neuronal differentiation through the CDK5 pathway in a Gs protein-independent signaling.

**Figure 3:**
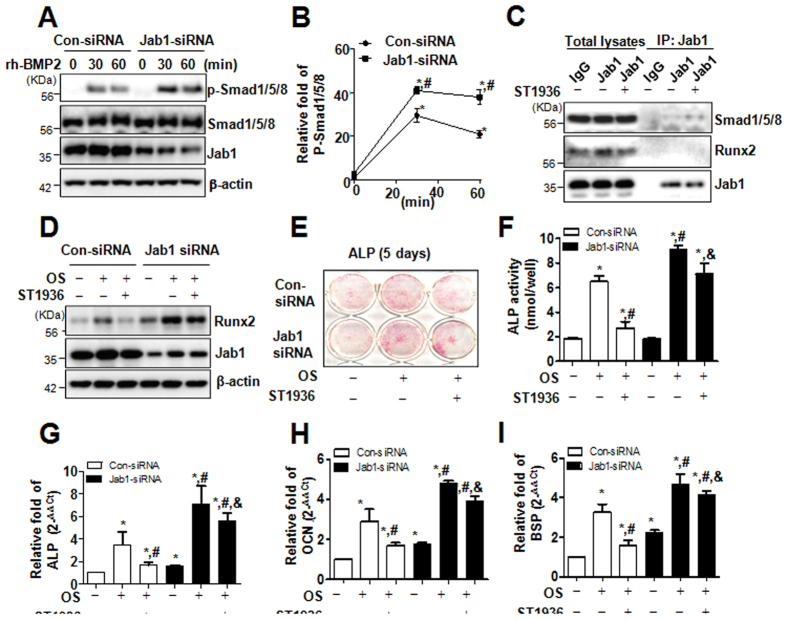
5-HT_6_R suppresses BMP-2 signaling by Jab1 to inhibit osteoblast differentiation. (**A,B**) Primary calvarial osteoblasts from 1-day-old ICR mice were transfected with negative control siRNA or Jab1 siRNA for 24 h, further incubated in serum-free medium for 24 h, and then treated with 100 ng/mL rh-BMP2 for the times indicated. The levels of p-Smad1/5/8, Smad1/5/8, Jab1, and β-actin were analyzed by Western blot (**A**). The line graph data are expressed as relative fold-changes from each control (**B**). (**C**) Cells were cultured in OS with ST1936 for 3 days, and were immunoprecipitated with an anti-mouse antibody and mouse anti-Jab1 antibody. The immune complexes and cell lysates were analyzed by immunoblotting with rabbit anti-Jab1, anti-Runx2, and anti-Smad1/5/8 antibodies. (**D**) After Cells were transfected with negative control siRNA or Jab1 siRNA for 24 h, the cells were cultured in OS with ST1936 for 3 days. Protein levels of Runx2, Jab1, and β-actin were analyzed by Western blot. (**E**,**F**) Cells were transfected with control siRNA or Jab1 siRNA for 24 h, and then cultured in OS with ST1936 for 5 days. ALP was evaluated via ALP staining (**E**) and ALP activity (**F**). (**G**–**I**) After Cells were transfected with negative control siRNA or Jab1 siRNA for 24 h, the cells were cultured in OS with 10 μM ST1936 for 7 days. The mRNA levels of ALP (**G**), OCN (**H**) and BSP (**I**) were analyzed by real-time PCR, and normalized to that of β-actin. Data shown represent the means ± SEM. *p < 0.05, vs. control. ^#^*p* < 0.05, vs. OS. &, *p* < 0.05, con-siRNA vs. Jab1 siRNA with 10 μM ST1936.

It was previously reported that 5-HT_6_R physically and functionally interacts with Jab1 and the activation of 5-HT_6_R releases Jab1^13^,^24^. Jab1 has been reported to interact with Smad5 and cause an attenuation of BMP-dependent transcriptional responses, suggesting that Jab1 might act as an inhibitor of BMP signaling^26^. Also, Chen *et al*., reported that knockout of Jab1 increased BMP-induced phosphorylation of Smad1/5 compared with wild-type controls, but not TGF-beta induced Smad2/3^27^. We, therefore, examined whether 5-HT_6_R modulates BMP2 signaling via Jab1 in primary osteoblasts. In the present study, the knockdown of endogenous Jab1 by RNA interference significantly enhanced rh-BMP-2-induced phosphorylation of Smad1/5/8, in a time-dependent manner (Fig. 3A,B), and the activation of 5-HT_6_R stimulated the binding of Jab1 with Smad1/5/8 (Fig. 3C). Moreover, the knockdown of Jab1 attenuated 5-HT_6_R agonist induced–Runx2 suppression (Fig. D) as well as osteoblast differentiation as evidenced by ALP staining (Fig. 3E) and activity (F) and differentiation markers, ALP, OCN, and bone sialoprotein (BSP) (Fig. 3G–I). These data suggest that 5-HT_6_R inhibits bone formation via Jab1-mediated BMP2 signaling.

**Figure 4:**
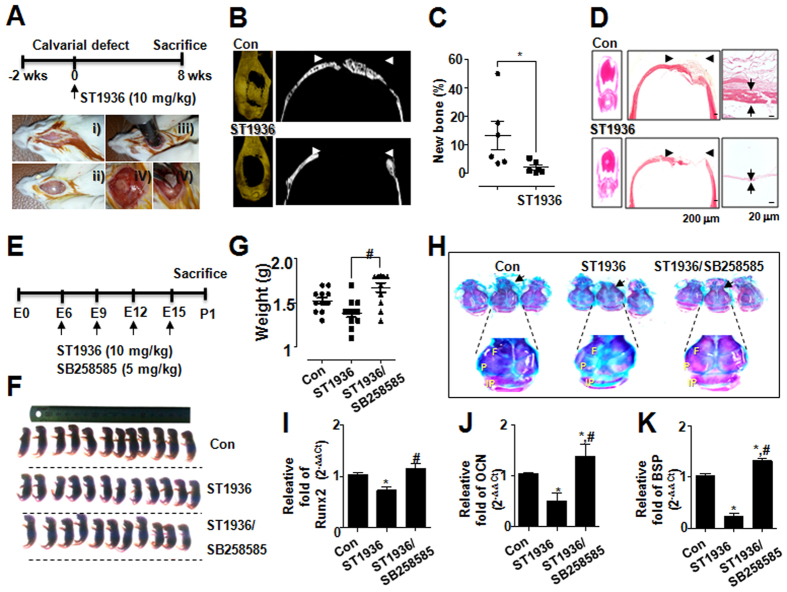
5-HT_6_R disrupted *in vivo* calvarial bone regeneration and embryonic calvarial bone development. (**A**) Scheme for assessing calvarial defect model. After female 8-week-old ICR mice were acclimated for 2 weeks, calvarial bone from female 10-week-old ICR mice was removed, and the calvarial defect was covered for 8 weeks with a bioabsorbable collagen sponge loaded with ST1936. **(B)** 3D micro CT (μCT) images from sham (control) and ST1936-treated groups were conducted to visualize and quantify calvarial bone regeneration. **(C)** Quantitative analysis of the relative percentage of new bone formation was performed by 3D μCT imaging and plotted as individual points. (**D**) H&E-stained images of the calvarial bone (*left*) and higher magnification images (*right*). Arrowhead indicates the defect region and arrows indicate width of bone tissue. Scale bars, 50 μm. (**E**) Scheme for assessing calvarial bone development by SR6. ST1936 (10 mg/kg, ip) or ST1936 (10 mg/kg, ip)/SB258585 (5 mg/kg, ip) were injected once per day at embryonic periods (**E**) 6, E9, E12, and E15. All mice had pups at E18 and postnatal 1 day (P1) mice were sacrificed. (**F,G**) P1 mice from each group were photographed (**F**) and weighed (**G**). (**H**) P1 mice were stained with Alizarin Red (red color) and Alcian Blue (blue color). The panels show calvarial bones consisting of paired frontal bones (**F**), paired parietal bones (**P**), and interparietal bone (IP). (**I**–**K**) The mRNA levels of Runx2 (**I**), OCN (**J**), BSP (**K**) in the calvarial bones of P1 mice were analyzed by real-time PCR and normalized to that of β-actin. Data shown represent the means ± SEM. *p < 0.05, vs. control. ^#^*p* < 0.05, vs. ST1936 alone.

The hypothesis that 5-HT_6_R inhibits bone formation via peripheral serotonin-mediated system was supported by the osteogenic effect of 5-HT_6_R in *in vivo* animal experiments (Fig. 4). To that end, we generated mice with surgery-generated calvarial defect, and bioabsorbable collagen sponge loaded with ST1936 (10 mg/kg) was placed into the defects (Fig. 4A). The local delivery of ST1936 into critical-sized mouse calvarial defects elicited an impaired osteogenic response (Fig. 4B–D). New bone formation visualized by 3D micro-CT images (μCT) was particularly decreased in defects treated with ST1936 (Fig. 4B) and the amount of new bone formation in the ST1936-treated group was significantly lower than that in the control group (*P* < 0.05) (Fig. 4C). Histological evaluation further supported the μCT findings, showing newly formed bone within the defect in controls, and no bone formation in the ST1936-treated group (Fig. 4D), indicating that 5-HT_6_R is required for osteoblast bone formation.

Calvarial sutures are major growth sites of intramembranous osteogenesis during craniofacial bone development^28^,^29^; thus, we further investigated whether 5-HT_6_R signaling has a critical role in intramembranous osteogenesis of calvarial bone and suture development. ST1936 or SB258585 was treated for the indicated embryonic period, and the phenotypes of bone in mice were analyzed (Fig. 4E–G). Sequential staining of mice skeletons revealed an increase in the ratio of cartilaginous (blue) to mineralized tissue (red) in the ST1936-adminstrated calvarial bones such as two frontal, two parietal, and an interparietal bone, whereas SB258585 administration reversed the effects of ST1936 (Fig. 4H). Such deformities of bones are mainly observed in calvarial sutures that are main centers for osteoblast differentiation and new bone formation postnatally^29^. We next examined osteoblast differentiation in calvarial bones by measuring the earliest molecular determinant of bone formation (Runx2) and late osteoblast differentiation markers (OCN and BSP)^30^. Our results showed that the 5-HT_6_R antagonist significantly reversed the 5-HT_6_R agonist–suppressed expression of Runx2 (Fig. 4I) and osteoblast-related genes, OCN and BSP (Fig. 4J,K). Taken together, these *in vivo* results demonstrated that 5-HT_6_R is a critical receptor to regulate bone formation in peripheral serotonin-mediated system.

In conclusion, these experiments have uncovered a novel link between peripheral serotonin system and 5-HT_6_R in bone formation. This is the first study to demonstrate that 5-HT_6_R is expressed in bone, which is crucial for *in vitro* osteoblast differentiation and *in vivo* bone development and regeneration in animal models via peripheral serotonin-mediated system. These findings suggest that 5-HT_6_R is a potential therapeutic target for bone repair and bone diseases, and will provide basic understanding on how peripheral serotonin-mediated system regulates bone formation.

## Materials and Methods

### Primary calvarial osteoblasts culture, differentiation, and transfection

Primary calvarial osteoblasts were isolated from calvariae of 1-day-old ICR mice using 0.2% collagenase-dispase enzyme solution (Sigma-Aldrich, St. Louis, MO). The cells isolated from the last four to six digests were cultured separately in α-modified minimum essential medium (α-MEM) (Gibco Laboratories, Grand Island, NY) containing 10% fetal bovine serum (FBS; Gibco Laboratories) and antibiotics (100 mg/mL penicillin G and 100 IU/mL streptomycin). After reaching a subconfluent state, the cells were removed from each flask and combined together as osteoblasts. The cells were used for all experiments at second passage, as described below. Cells were cultured in α-MEM containing 10% FBS, 5 mmol/L β-glycerophosphate, ascorbic acid (50 μg/mL), and antibiotics. For siRNA transfection, cells were transfected using Lipofectamine RNAiMAX according to the manufacturer’s specification (Invitrogen, Carlsbad, CA, USA).

### Mice and ethics statement

Female 8-week-old ICR mice (Samtako, Osan, Kyoung Gi-Do, Korea) used in this study were maintained in accordance with the National Institute of Toxicological Research of the Korea Food and Drug Administration guidelines for the humane care and use of laboratory animals. All experimental procedures in the current study were approved by Kyung Hee University Animal Care Committee (approval number: KHMC-IACUC 2015-002). All experimental methods were conducted in accordance with relevant guidelines.

### Calvarial bone defects

The mice were anesthetized, and a 5-mm-diameter calvarial critical-sized defect was created on each side of the calvarial bone using a dental bur attached to a slow-speed hand piece with minimal invasion of the Dura mater. The wound was closed with CollaTape (Integra LifeSciences Corporation, Carlsbad, CA) containing ST1936 (10 mg/kg, Tocris Bioscience, Bristol, United Kingdom). Animals were sacrificed 8 weeks postsurgery and the calvarial bone was carefully excised, cleaned, and fixed immediately in 10% formalin. After imaging by micro-computerized tomography (μCT), tissues were demineralized in10% EDTA for 14 days, embedded in paraffin, and sectioned at 5 μm. Sections were stained with hematoxylin and eosin (H&E).

### Micro-computed tomography (μCT)

μCT was performed at the Advanced Institutes of Convergence Technology (Genoss Co., Ltd., Gyeonggi-do Korea). Micro-CT data of calvaria were acquired on a Skyscan 1173 scanner (Bruker-microCT, Kontich, Belgium). Scanning was carried out at 75 kV/106 μA for 500 ms. In total, 800 projections were collected at a resolution of 9.94 μm/pixel. Reconstruction of sections was carried out with software associated with the scanner (Nrecon), with the beam hardening correction set to 40%. Realistic 3D-Visulizatio software (Bruker-microCT, Konitch, Belgium) was used to reconstruct the CT images three-dimensionally, which were acquired on approximately 2,000 cross-sections.

### Calvarial bone development and skeletal staining

Mice received intraperitoneal injection of ST1936 (10 mg/kg, Tocris Bioscience) or SB258585 (5 mg/kg, Tocris Bioscience) four times at E6, E9, E12, E15. Briefly, newborn mice were deskinned, eviscerated, and immersed in 100% ethanol for 24 h. The samples were fixed in acetone for 24 h and then stained for 24 h in a solution containing 0.1% Alizarin Red, 0.3% Alcian Blue, acetic acid, and 70% ethanol (1:1:1:17, v/v/v/v). They were then transferred to a solution of 1% KOH in 20% glycerol until clear and then stored in glycerol.

### ALP activity

ALP activity was measured by spectrophotometry. Cells were homogenized in 0.5 mL of distilled water using a sonicator, and centrifuged. Aliquots of cell homogenate were incubated with 15 mm *p*-NPP in 0.1 m glycine-NaOH (pH 10.3) at 37 °C for 30 min. The reaction was stopped by the addition of 0.25 n NaOH. The absorbance was measured at 405 nm using an ELISA reader (Beckman Coulter).

### ALP staining

Cells were washed with 1× PBS and then fixed in 4% formaldehyde for 20 min at room temperature. The cells were rinsed with distilled water, and permeabilized with 0.1% Triton X-100. Cells were incubated at 37 °C for 30 min in Naphthol-AS-BL alkaline solution mixture (Sigma-Aldrich).

### Alizarin Red S staining

After 14 days of culture, cells were fixed in 70% ice-cold ethanol for 1 h and rinsed with distilled water. Cells were stained with 40 mM Alizarin Red S (pH 4.2) for 10 min with gentle agitation. The level of Alizarin Red S staining was observed under light microscopy. To quantify Alizarin Red staining, stains were eluted with 100% DMSO and measured at 590 nm.

### Reverse transcriptase-polymerase chain reaction (RT-PCR) and quantitative real-time PCR

The total RNA of cells was extracted using TRIzol™ reagent (Life Technologies, Gaithersburg, MD) according to the manufacturer’s instructions. RNA (1 μg) isolated from each sample was reverse-transcribed using oligo (dT)_15_ primers with AccuPower^®^ RT PreMix (iNtRON Biotechnology, Gyeonggi-do, South Korea). Next, the generated cDNAs were amplified with AccuPower^®^ PCR PreMix (Bioneer Corporation, Daejeon, South Korea). The primer sequences are as follows:

ALPF: 5′-ACACCTTGACTGTGGTTACTG-3′, R: 5′-CCATATAGGATGGCCGTGAAG-3′

Runx2F: 5′-ACTCTTCTGGAGCCGTTTATG-3′, R: 5′-GTGAATCTGGCCATGTTTGTG-3′

OCNF: 5′-ACACCATGAGGACCATCTTTC-3′, R:5′-CGGAGTCTGTTCACTACCTTATT-3′

BSPF: 5′-TGTTTGTAGTGGGCTTCTTCTT-3′, R: 5′-TCCATCTAGTCCCAGCTCATAG-3

5-HT1RF: 5′-TCCACTCACCTCTCACAGTAT-3′, R: 5′-CTCACACCCACACTTCCTTAG-3′

5-HT2RF: 5′-TCACCATTGCGGGAAACA-3′, R : 5′-AGGAAACCCAGCAGCATATC-3′

5-HT3RF: 5′- CTCGCTGAGACCATCTTCATT-3′, R : 5′-ATCCAGGCTATTCTGTCTAGGA-3′

5-HT4RF: 5′-GCCTTGTCACTCTTGCTATCT-3′, R: 5′-TACATTTGGGTCCTCTGACTTG-3′

5-HT5RF: 5′-CTGTGCTGACTTCTCCCATAAA-3′, R: 5′-GCTGAGAACCACATGCTAAGA-3′

5-HT6RF: 5′-CCGTATGTGACTGCATCTCTC-3′, R: 5′-ATGATAGGGTTCATGGTGCTATT-3′

5-HT7RF: 5′-GCTGAGACTGCACAACAGAA-3′, R: 5′-GTTGCCATCTCCCTCAAGATAC-3′

β-actinF: 5′-AATGTGGCTGAGGACTTTG-3′, R: 5′-GGGACTTCCTGTAACCACTTATT-3′

For mRNA quantification, total RNA was extracted using the RNAqueous^®^ kit and the cDNA was synthesized using 1 μg of total RNA with the High Capacity RNA-to-cDNA kit (Applied Biosystems, Foster City, CA) according to the manufacturer’s protocol. Quantitative real-time PCR was performed using a LightCycler^®^ 1.5 System (Roche Diagnostics GmbH, Mannheim, Germany). Thermocycling conditions consisted of an initial denaturation of 10 s at 95 °C, followed by 45 cycles of 95 °C for 10 s, 60 °C for 5 s and 72 °C for 10 s. For the calculation of relative quantification, the 2^−ΔΔ*C*T^ formula was used, where –ΔΔ*C*T = (*C*_T,target_ − *C*_T,β-actin_) experimental sample – (*C*_T,target_ − *C*_T,β-actin_) control sample.

### Western blot analysis

Cells were washed twice with ice-cold PBS, and lysed in 20 mM Tris-HCl buffer (pH 7.4) containing a protease inhibitor mixture (0.1 mM PMSF, 5 mg/mL aprotinin, 5 mg/mL pepstatin A, and 1 mg/mL chymostatin). Protein concentration was determined using Bradford reagent (Bio-Rad, Hercules, CA). Equal amounts of lysates (20 μg) resolved by sodium dodecyl-polyacrylamide gel electrophoresis (SDS–PAGE) were transferred to a polyvinylidene fluoride (PVDF) membrane (Millipore, Bedford, MA), and the membrane was blocked with 1× TBS containing 0.05% Tween 20 and 5% skim milk or 2% BSA for 1 h at room temperature. After blocking, the membranes were incubated overnight at 4 °C with the respective primary antibodies, as follows: p-ERK1/2 (1:2000, #9101S, Cell Signaling Technology, Beverly, MA), ERK1/2 (1:2000, #9102S, Cell Signaling), Runx2 (O1L7F) (1:1000, #12556S, Cell Signaling), Jab1 (1:2000, #6895, Cell Signaling), p-Smad1/5/8 (D5B10) (1:2000, #13820S, Cell Signaling), Smad1/5/8 (N-18) (1:1000, #sc-6031-R, Santa Cruz Biotechnology, Santa Cruz, CA), β-actin (C4) (1:1000, #sc-47778, Santa Cruz Biotechnology). The membranes were washed with 1× PBS and incubated with diluted horseradish peroxidase (HRP)-conjugated secondary antibodies (1:10,000, Jackson ImmunoResearch, West Grove, PA) for 1 h at room temperature. After three washes, the membranes were detected using an enhanced chemiluminescence (ECL) kit (Millipore, Bedford, MA).

### Co-immunoprecipitation

Cells and tissues were gently lysed with lysis buffer for 1 h on ice and then centrifuged at 15,000 g and 4 °C for 15 min, and the supernatant was collected. The soluble lysates were incubated with mouse anti-Jab1 antibody (#sc-13157, Santa Cruz Biotechnology) at 4 °C, and then with Protein A/G bead (Santa Cruz Biotechnology) and washed times. Immune complexes were eluted by boiling for 10 min at 95 °C in SDS sample buffer, followed by Western blot analysis with anti-Jab1 (1:1000, Cell Signaling), anti-Runx2 (1:1000; Cell Signaling Technology), or anti-Smad1/5/8 (1:1000; Santa Cruz Biotechnology) antibodies.

### Statistical analysis

The data were analyzed using GraphPad Prism version 5 software (GraphPad Software, Inc., San Diego, CA). Data are presented as the means ± S.E.M. Statistical significance was evaluated using one-way analysis of variance (ANOVA) and the differences were assessed by the Dunnett’s test. A value of P < 0.05 was considered to indicate statistical significance.

## Additional Information

**How to cite this article**: Yun, H.-M. *et al*. Peripheral serotonin-mediated system suppresses bone development and regeneration via serotonin 6 G-protein-coupled receptor. *Sci. Rep.* **6**, 30985; doi: 10.1038/srep30985 (2016).
